# Altered Red Blood Cell Membrane Fatty Acid Profile in Cancer Patients

**DOI:** 10.3390/nu10121853

**Published:** 2018-12-01

**Authors:** Javier Amézaga, Sara Arranz, Ander Urruticoechea, Gurutze Ugartemendia, Aitziber Larraioz, Maria Louka, Matxalen Uriarte, Carla Ferreri, Itziar Tueros

**Affiliations:** 1 AZTI, Food and Health, Parque Tecnológico de Bizkaia, Astondo Bidea, 609, 48160 Derio, Bizkaia, Spain; jamezaga@azti.es (J.A.); sarranz@azti.es (S.A.); mauriarte@azti.es (M.U.); 2 Onkologikoa Foundation, Paseo Doctor Begiristain, 121, 20014 San Sebastián, Gipuzkoa, Spain; anderu@onkologikoa.org (A.U.); gugartemendia@onkologikoa.org (G.U.); alarraioz@onkologikoa.org (A.L.); 3 Lipinutragen, Via di Corticella, 181/4, 40128 Bologna, Italy; maria.louka@lipinutragen.it; 4 ISOF, Consiglio Nazionale delle Ricerche, Via Piero Gobetti, 101, 40129 Bologna, Italy

**Keywords:** cancer, membrane lipidome, red blood cell, unsaturated fatty acids, saturation index, desaturase index

## Abstract

The fatty acid (FA) composition of red blood cell (RBC) membrane phospholipids of cancer patients can reflect tumor status, dietary intakes, and cancer type or therapy. However, the characteristic membrane profiles have so far not yet defined as a potential biomarker to monitor disease evolution. The present work provides the first evidence of cancer metabolic signatures affecting cell membranes that are independent of nutritional habits. From the Oncology Outpatient Unit of the Onkologikoa hospital, two groups of cancer patients (*n* = 54) and healthy controls (*n* = 37) were recruited, and mature RBCs membrane phospholipids were analyzed for FA profiling (GC-MS). Dietary habits were evaluated using a validated food frequency questionnaire. The adjusted Analysis of Covariance Test (ANCOVA) model revealed cancer patients to have a lower relative percentage of saturated fatty acids (SFA) (C16:0 (5.7%); C18:0 (15.9%)), and higher monounsaturated fatty acids (MUFA) (9c-C18:1 (12.9%) and 11c-C18:1 (54.5%)), compared to controls. In line with this, we observe that the desaturase enzymatic index (delta-9 desaturase (Δ9D), +28.3%) and the membrane saturation index (SI = SFA/MUFA; −27.3%) were similarly modulated. Polyunsaturated fatty acids (PUFA) families showed an increase of *n*-6 C18:2 and C20:3 (15.7% and 22.2% respectively), with no differences in *n*-6 C20:4 and *n*-3 PUFA (docosahexaenoic acid (DHA) and eicosapentaenoic acid (EPA)). Importantly, these changes were found independent of foods and fat intakes from the diet. The membrane lipid profile in RBC was useful to ascertain the presence of two main metabolic signatures of increased desaturation activity and omega-6 in cancer patients, statistically independent from dietary habits.

## 1. Introduction

Cancer is one of the leading causes of morbidity and mortality in our society, being responsible for nearly one sixth of deaths and is expected to rise by about 70% over the next two decades [1]. In the last years, many epidemiological studies related to the assessment of the relationship between nutrition and cancer have been done [2]. However, studies which focus on the role of nutrition for the oncological patient are less frequent. It is commonly agreed that a poor nutritional status during cancer treatment weakens the patient, renders the administration of an appropriated treatment difficult, and increases treatment side-effects [3]. Even the prognosis for cancer patients with weight loss is worse than that for weight stable patients [4]. There are several guidelines providing nutritional recommendations for cancer patients during the disease, mainly addressed to minimize treatment side effects. Despite the increasing demand for specific nutritional recommendations during the disease, tools for personalized nutritional indications and nutrition support are not yet used routinely as an adjunct to treatments, and only if there is evidence of some nutritional risk or when enteral or parenteral nutrition are exclusive.

Recent directions suggested that molecular nutrition may give insights about the specific nutritional requirements in cancer patients according to their metabolism and the impact of a precise diet [5]. Regarding different nutrients, the interest on lipids has been increased in recent years, due to their function in constant modulation to meet the cellular needs. Lipid metabolic reprogramming is involved in several human diseases, including metabolic, immune, and central nervous system disorders, and is now firmly established as a hallmark of cancer [6]. Indeed, in addition to the synthesis of DNA and proteins, the production of lipids is necessary for cell growth and proliferation. Saturated and monounsaturated fatty acids (SFA and MUFA) make part of the *de novo* biosynthesis, involving the enzymatic complex of fatty acid synthase (FASN) and the activity of delta-9 desaturase (Δ9D, stearoyl-CoA desaturase, SCD-1), and both enzymes are overexpressed in cancer, attracting interest for their inhibition [7,8]. It is worth noting that there is a debate on cancer cell requirement for endogenously synthesized fatty acids or the same fatty acids obtained from the diet, since some reports did not find differences in these two origins [9]. Untargeted/targeted lipidomics studies have clarified the crucial role of lipid classes and molecular species in supporting tumor growth and metastatic dissemination, thus envisaging new therapeutic targets disturbing lipid raft organization and improving apoptosis signaling [10]. The role of polyunsaturated fatty acids (PUFA) is intriguing, since human cells, including cancer cells, cannot reproduce and grow without them, and essential PUFA omega-6 and omega-3 fatty acids are matters of discussion for their effects in cancer incidence and evolution [11,12,13]. In this context, it is also worth mentioning that cell membranes are an interesting observational point for two reasons: (a) PUFA incorporation in membrane phospholipids and their release by the action of phospholipase A2 (PLA2) in order to start lipid signaling with formation of eicosanoids and other lipid mediators (i.e., the mitogenic prostaglandin E2) [14]; (b) the involvement of phospholipid fatty acid residues in the naturally occurring process of membrane remodeling, known as Lands’s cycle [15] and in such step the cellular pool of fatty acids, equilibrated from dietary and metabolic contributions, becomes a relevant determinant, inspiring the “membrane lipid therapy” as a tool for controlling the membrane lipidome properties and functions during diseases and aging [16]. Although FAs and their dietary intakes have been found to correlate with metabolic status [17,18,19], epidemiological or intervention studies considering dietary factors can be challenging to interpret, because of lack of personalization and the complexity of food and eating patterns.

Here, we are interested in membrane lipidomics related to phospholipids and FA as their hydrophobic components and we have developed the membrane fatty acid analysis based on a fatty acid cluster to get a molecular profile to be implemented in health and diseases for personalized nutrilipidomic approach [20]. We consider red blood cell (RBC) membrane as an ideal site for fatty acid evaluation, obtained by a non-invasive technique, as well as after inexpensive methodologies for extraction and analysis. The RBC membrane FA composition is representative of all FA families and of the general condition of other tissues from the body. It results from the interaction among genetic, metabolic and dietary factors. Therefore, the RBC membrane FA composition represents a comprehensive biomarker of the homeostatic condition of an individual. Several research groups have studied the effect of lipid alteration in the development of cancer disease with RBC membrane as a potential biomarker in various forms of the illness, such as breast, prostate, liver, lung and colorectal, evidencing interesting differences in several membrane FA levels [21,22,23,24]. Nonetheless, these studies did not examine in detail the dietary intake correlations on the RBC membrane lipid profile, therefore a step forward must be done by combining lipidome data with food and dietary assessment by food frequency questionnaire (FFQ) in a statistical treatment. 

Based on the above premises, we planned an exploratory study using the mature erythrocyte for its fatty acid content expressing the four months lifetime in circulation throughout all body tissues [25]. We used a high-throughput procedure to isolate and process this cell type which allows this information to be gathered with high fidelity, as reported in previous studies [26,27,28]. We also used a validated FFQ and careful patient interviews to collect all relevant dietary data and applied a robust statistic treatment to examine the results.

The aim of this study is to examine differences in the lipid profile of mature RBC membranes of cancer patients and healthy controls, by controlling for dietary habits, and finally to foster further interest in membrane fatty acids as a biomarker for personalized treatments in cancer patients.

## 2. Materials and Methods 

### 2.1. Subjects and Study Design

A prospective observational study was carried out on a group of adult patients undergoing chemotherapy (CT) at the Oncology Outpatient Unit from Onkologikoa Foundation (San Sebastian, Spain). Potentially eligible patients were identified from January 2015 to May 2015 by nurses during the outpatient consultation. A subsample of patients, from a previous study, was recruited [29]. Inclusion criteria included patients aged between 18 and 70 years old, receiving a chemotherapy regimen for the neoadjuvant, adjuvant or metastatic treatment of any cancer (except head and neck cancer considering previous study criteria [29]), and Body Mass Index (BMI) < 25. The healthy control group consisted of healthy subjects from Onkologikoa Hospital, matched for BMI, age and gender. Those having suffered cancer in the last five years were excluded.

The study protocol was approved by the Gipuzkoa Clinical Research Ethics Committee (TUE-SEN-2014-01) and accomplished according to the Declaration of Helsinki Good Clinical Practice guidelines. Written informed consent was obtained from all patients.

### 2.2. Nutritional Status and Dietary Intake

Weight and height were recorded by nurses from Onkologikoa. Body Mass Index (BMI) was assessed according to the World Health Organization criteria. Dietary intake was evaluated using a 137-item food frequency questionnaire (FFQ), validated for the Spanish population [30] and were referred to the last 4–6 months. Two trained researchers completed FFQ with patients within 20–25 min. personal interviews during their visits to the Outpatient Unit for CT treatment. The nutrient composition of their diets was determined using DIAL software (UCM & Alce Ingeniería S.A, Madrid, Spain) (V 3.4.0.10) [31].

### 2.3. Red Blood Cell Membrane Fatty Acid Profile Analysis

The fatty acid composition of mature RBC membrane phospholipids was obtained from venous blood samples (approximately 2 mL) collected in vacutainer tubes containing ethylenediaminetetraacetic acid (EDTA) in the fasting state. Samples were stored at 4 °C until analysis. Blood work-up for lipid extraction and lipid transesterification to fatty acid methyl esters (FAMEs) was performed using an automated protocol, which included a selection of mature RBCs as previously reported [26,27,28] and detailed below. Briefly, the whole blood in EDTA was centrifuged (4000 rpm for 5 min at 4 °C), and the samples entered an automatized procedure for mature RBCs selection. The cell fraction was isolated on the basis of high density of the aged cells [32] and was standardized according to the cell diameter which is reduced compared to the general population as determined by Scepter cell counter counts (Scepter 2.0, EMD Millipore, Darmstadt, Germany). The robotics performs all the subsequent steps for the cell lysis, isolation of the membrane pellets, phospholipid extraction from pellets using the Bligh and Dyer method [33], transesterification to FAMEs by treatment with a potassium hydroxide (KOH)/methyl alcohol (MeOH) solution (0.5 mol/L) for 10 min at room temperature and extraction using *n*-hexane (2 mL). FAMEs were analyzed using capillary column gas chromatography (GC). GC analysis was run on the Agilent 6850 Network GC System, equipped with a fused silica capillary column Agilent DB23 (60 m × 0.25 mm × 0.25 μm) and a flame ionisation detector, using published conditions. Optimal separation of all fatty acids and their geometrical and positional isomers is achieved Identification was made by comparing them to commercially available standards and to a library of trans isomers of monounsaturated fatty acids (MUFAs) and polyunsaturated fatty acids (PUFAs) as previously described [26,27,28]. The amount of each FA was calculated as a percentage of the total FA content (relative %), being > 97% of the GC peaks recognized with appropriate standards.

### 2.4. Membrane Fatty Acid Cluster

The fatty acid (FA) panel considered 12 fatty acids, representative of the main building blocks of the RBC membrane glycerophospholipids and of the three FA families: SFAs, palmitic acid (C16:0); stearic acid (C18:0); MUFAs, palmitoleic acid (C16:1;9c); oleic acid (C18:1; 9c); cis-vaccenic acid (C18:1; 11c); *n-*3 PUFAs, (eicosapentaenoic acid (EPA): C20:5; docosahexaenoic acid (DHA): C20:6); *n-*6 PUFAs, linoleic acid (LA): C18:2; dihomo-gamma-linolenic acid (DGLA): C20:3; arachidonic acid (AA): C20:4; trans isomers, considering elaidic acid (C18: 1; 9t) and mono-trans arachidonic acid isomers (mono trans-C20:4; *n-*6). 

Considering these fatty acids, different indexes were calculated [34]: Membrane fluidity index or saturation index (SI) (%SFA/%MUFA), inflammatory risk index (%Omega 6/%Omega 3), cardiovascular risk index (%EPA + %DHA) [35], PUFA balance ((%EPA + %DHA)/total PUFA × 100) [36], Free radical stress index (sum of trans-18:1 + Σ monotrans 20:4 isomers), Unsaturation Index (UI) ((%MUFA × 1) + (%LA × 2) + (%DGLA × 3) + (%AA × 4) + (% EPA × 5) + (%DHA × 6), and Peroxidation Index (PI) ((%MUFA × 0.025) + (%LA × 1) + (%DGLA × 2) + (%AA × 4) + (% EPA × 6) + (%DHA × 8)). Additionally, the enzymatic indexes of elongase and desaturase enzymes, the two classes of enzymes of the MUFA and PUFA biosynthetic pathways, were estimated by calculating the product/precursor ratio of the involved FAs.

### 2.5. Statistical Analysis

Differences between groups for the population characteristics (BMI, age), nutrient intake and unadjusted FA levels were tested with U Mann-Whitney test for those data that were not normally distributed, two tailed t-Test for normally distributed variables and Chi-square test for the categorical variable (gender). Normal data distribution was verified using a Kolmogorov-Smirnov test. In addition, the FA levels from both groups were compared with Analysis of Covariance Test (ANCOVA), adjusting for age, nutrient intake and BMI. A Principal Component Analysis (PCA) was conducted on nutrient intake variables to reduce and simplify the dimension of these variables. Generated components were rotated by an oblique rotation (direct oblimin) to increase interpretability. The Kaiser-Meyer-Olkin (KMO) and Bartlett’s test of sphericity were used to verify the sampling adequacy for the analysis. With an eigenvalue cut-off > 1, component interpretability and screen plot were used to decide the number of factors to retain. These components were included in the ANCOVA analysis. The level of significance was set at *p* < 0.05. All statistical analyses were performed using SPSS (IBM Corp. V 24.0, New York, USA).

## 3. Results

### 3.1. Population Characteristics

54 cancer patients and 37 controls were included in the study. The socio-demographic characteristics of this sample are shown in Table 1. Age is reported for each group as the median and first and third quartiles. BMI is presented as mean and standard deviation (SD) and cancer type and gender as the number of cases and the percentage. There were no differences in gender distribution and BMI between groups, as they were matched. Cancer group was older (51–64 years) compared to the control group (32–57 years). The age difference was statistically significant (*p* < 0.001).

The most predominant cancer type was breast (37%) followed by colon (24%) and lung (17%). Patients mainly were receiving a metastatic treatment (63%), but there were also patients undergoing neoadjuvant (30%) and adjuvant therapies (7%).

### 3.2. Dietary Intake

The information of dietary intake, including nutrients and food categories, is shown in Table 2. There were some differences between cancer and control groups according to dietary intake for individual nutrients and for food categories. Cancer group was characterized by a diet richer in oily and lean fish (*p* < 0.001 and *p* = 0.001 respectively), olive oil (*p* < 0.001) and dairy products (*p* < 0.001) than controls. There were no differences in the intake of shellfish, nuts, fruits and vegetables, eggs and red meat between groups. Considering the nutrient composition of the diets, there were no statistically significant differences in energy intake, carbohydrates and proteins. Cancer patients had a lower intake of simple sugars (*p* < 0.001), fiber (*p* < 0.001) and alcohol (*p* < 0.001); and a higher intake of fats (*p* = 0.001), more specifically, total MUFA (*p* = 0.011) and total PUFA (*p* = 0.007). Considering specific fatty acids, there were no differences in C16:1; 9c and C18:1; 9c intake. With regard to SFA, C16:0 (*p* = 0.004) and C18:0 (*p* < 0.001) intake levels were lower in the cancer group and no differences were found for C14:0 intake. Regarding PUFAs, *n-*3 C18:3 (*p* < 0.001), *n-*3 C20:5 (*p* < 0.001), *n-*3 C22:5 (*p* < 0.001), *n-*3 C22:6 (*p* < 0.001) and *n-*6 C20:4 (*p* = 0.024) intake levels were higher in the cancer group. 

### 3.3. Red blood cell Membrane Fatty Acid Profile

As described in the Materials and Methods section, the membrane fatty acid profile of the subjects was based on a fatty acid cluster of ten cis fatty acids and two trans fatty acid isomers expressed as relative percentage of the cluster, proposed in the approach of fatty acid-based functional lipidomics taking into account the most representative fatty acid families and their roles in the membrane properties [20]. From these values the lipid values and indexes were calculated before and after ANCOVA adjustment and are shown for each group in Table 3. This adjustment controlled the effect of other factors, such as age, BMI and food intake. To ease the data analysis before ANCOVA, the information relative to food intake was simplified using a previous PCA analysis, where different models with various combinations of nutrients were tested. Finally, we carried out a three factor PCA model, including 14 items. The model KMO (0.71) and KMO values for individual items (> 0.61) indicated a good sampling adequacy for the analysis [37]. Selected factors explained 80.93% of the variance in the PCA analysis. ANCOVA test provided adjusted means but in three cases (Total SFA, Total Trans and Saturation Index), the p-values were not calculated because their data did not meet the Levene test conditions.

FA levels in RBC membrane in the unadjusted and adjusted model are slightly different. The effect of the controlling factors in the ANCOVA adjusted model (nutrient intake, BMI and age) was especially remarkable in PUFA levels. In the unadjusted model there were statistically significant differences for C20:4 (*p* = 0.033) and C20:5 (*p* = 0.026) between groups, whereas in the adjusted model these differences disappeared. On the contrary, in the unadjusted model there were no differences in the RBC linoleic acid (LA) levels between groups, but after adjustment, there is a statistically significant difference, with increasing LA levels in the cancer group. For the other FA belonging to the MUFA and SFA families, there were no changes after adjustment.

The regression model analysis revealed several differences between cancer and control mature RBC lipid profiles. Cancer patients presented a lower relative percentage of SFA, higher levels of total MUFA, and higher levels of total PUFA compared to the control group. Considering specific fatty acids, C16:0 and C18:0 were lower in the cancer group (5.7% and 15.9% respectively), whereas C18:1; 9c and C18:1; 11c were higher (12.9% and 54.5% respectively). Therefore, cancer patients had lower saturation index (SFA/MUFA) than controls (27.3%). On the other hand, PUFA *n-*6 C18:2 and *n-*6 C20:3 showed higher percentages in cancer patients (15.7% and 22.2% respectively) and no differences in *n-*6 C20:4 and *n-*3 PUFA (DHA and EPA) were found. Unsaturation index was higher in cancer patients (3.83%), No differences were found for peroxidation index. With regard to trans fatty acids (TFA), cancer patients have a less relative percentage of trans isomers trans-18:1 and trans-20:4 than controls. 

With respect to the enzymatic activity, calculated indirectly by difference between substrate and product, it must be highlighted that Δ9D presented a higher relative activity (28.3%), but only in the conversion between stearic/oleic acid, whereas Δ5D results in lower values in cancer patients compared to controls (23.0%) corresponding to a significant increase of the dihomo-gamma-linolenic acid values. 

No significances were found considering patients with different cancer type, chemotherapy regimen and treatment objective (adjuvant, neoadjuvant or metastatic) compared to the control group.

## 4. Discussion

**Figure 1:**
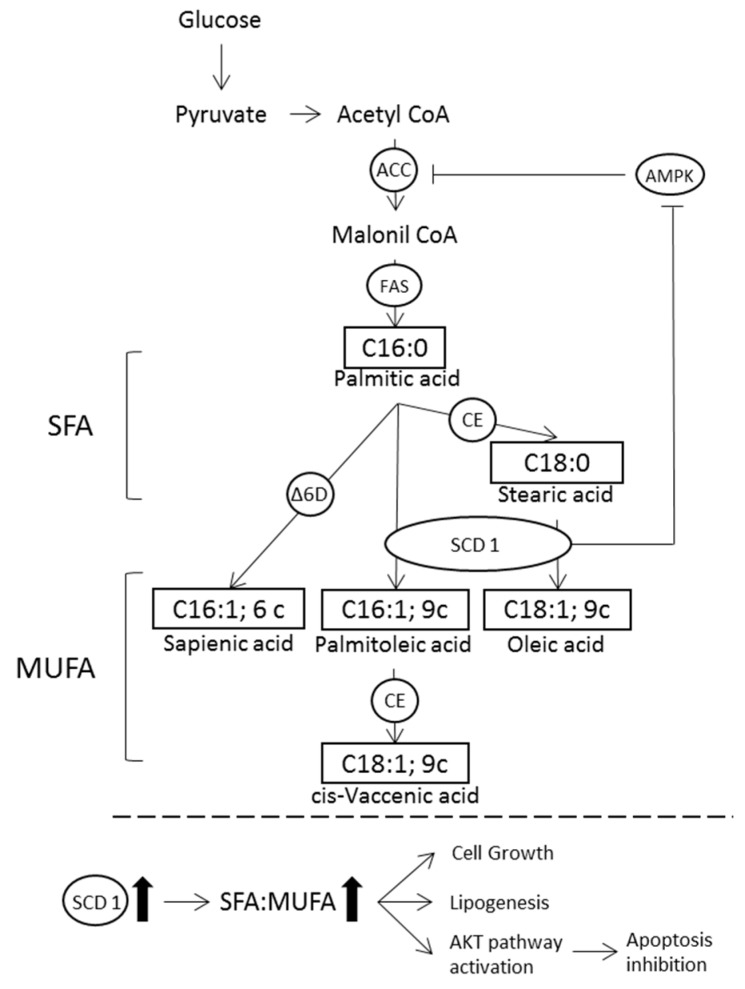
Mechanism for the control of *de novo* synthesis and lipid metabolism in cancer cells by SCD1. A high activity of SCD1 promotes the conversion of SFA to MUFA and keeps lipogenesis active by modulating potential mechanisms: (i) Maintaining ACC in an activated state by reducing levels of SFA, which are allosteric inhibitors of ACC; (ii) inactivating the AMPK (5’ adenosin monophosphate-activated protein kinase), which promotes the inactivation of ACC. SCD1 also plays a key role in enhancing the mitogenic and tumorigenic capacity of cancer cells. ACC, acetyl CoA carboxylase; FAS, fatty acid synthase, CE, 2-carbon chain elongation; Δ6D, delta-6-desaturase; SCD1, stearoyl CoA desaturase-1; SFA, saturated fatty acid; MUFA, monounsaturated fatty acid.

The complexity of metabolic changes due to cancer involves the metabolism of carbohydrates, proteins and lipids in order to enhance tumor cell proliferation. Non-diseased cells regulate their anabolism and catabolism depending on nutrient accessibility and physical activity, whereas cancer cells show unregulated growth regardless of availability or energy requirements, providing anabolic precursors to maintain protein and nucleic acid biosynthesis and membrane biogenesis [38,39]. The relevance of lipid *de novo* synthesis has been highlighted [7,8], cancer cells presenting an increased level of lipogenesis and a dependence of unsaturated FA for survival under unfavorable conditions. Membrane fatty acid composition expresses the dietary/metabolic balance in a tissue, but in cancer it was not known if statistical correlations occur with dietary habits. Therefore, a cohort of fatty acids representing the main fatty acid families of mature RBC membrane phospholipids in a group of cancer patients, having different tumor types and treatments, was for the first time evaluated with their food and fat intakes gathered by a validated FFQ. In such evaluation the homogeneity of the cohort for the tumor type or treatment was not considered, also because no cancer phenotype was determined. For clarity, we underline that under our analytical conditions, a specific class of glycerophospholipids with fatty acids esterified in the positions 2 and 3 of the glycerol moiety is considered, which is the “backbone” of cell membranes, with very well-known biochemical pathways and biological roles regulating fluidity and permeability properties. We found lower levels of SFA and higher levels of MUFA in cancer patients compared to the control group with higher levels of oleic acid (C18:1; 9c) and cis-vaccenic acid (C18:1; 11c). Considering that oleic acid is commonly found in this group’s diet, it is important to have eliminated the food influence on these parameters, and their significance indicates the balance of saturation and desaturation of cell membrane as a metabolic transformation, that not only provides cells with properties, such as permeability and fluidity, but also does not contribute to the increase of peroxidation and cellular damage [40]. It is worth recalling that *de novo* biosynthesis of palmitic and stearic acid as saturated fats must be accompanied by the desaturation step, otherwise saturated fats can compromise the correct function of the cell and its survival, because of their contribution to poor membrane permeability and fluidity [41]. The pathway to form oleic acid (C18:1; 9c) from stearic acid (C18:0) is activated in our cancer group, as we can see from the significant value of the corresponding index of the Stearoyl Coenzime A desaturase 1 (SCD1) activity for the higher conversion of SFA to MUFA. On the other hand, no statistically relevant increase is seen in the parallel desaturase transformation of palmitic (C16:0) to palmitoleic acid (C16:1;9c). The increase of desaturase transformation is well known in cancer [38,42] and, as shown in Figure 1, palmitic acid (C16:0) is at the cross road to form stearic acid (C18:0) after elongation and to produce oleic acid (C18:1; 9c), or to be directly desaturated to palmitoleic acid (C16:1,9c) and then elongated to cis vaccenic acid (C18:1; 11c). 

Cis-vaccenic acid presented the highest difference between groups, however, there is little information about this FA in cancer metabolism. Previous studies described the same pattern, where SFA levels are lower because the production of MUFA is prioritized and underlined the necessity of the cell to find a more fluid state and guarantee the cell survival [43,44,45]. The prognostic value of the increased content of MUFA was already underlined [21,23,38,46], some authors proposing that this difference in the saturation index are dependent with the cancer type [47]. Due to the low number of cancer types in our exploratory study, we cannot make such a differentiation. It is worth highlighting that in our cohort we ascertained that the increases of SCD1 index and levels of oleic acid and cis-vaccenic acid are independent of the cancer type or therapeutic treatment. Our results agreed with other studies for SFA and MUFA levels in RBC membrane, where the same pattern of high oleic acid and low stearic acid was reported for breast cancer [23], and with several cancer types (breast, prostate, liver, pancreas, colon and lung) studied together, as the present study [21]. It is also interesting to note that the SFA/MUFA pathway is also evoked in obesity [27,48], however, in such membrane fatty acid profiles it was observed the increase of palmitoleic acid, which is not present in our tumour patients. In obesity palmitoleic acid is an important biomarker of the metabolic contribution in RBC membranes, as it does not occur in a relevant amount in foods. This can be connected to the type of tissue involved in the disease, with MUFA accumulation in the adipose tissue as the most relevant compartment involved in obesity, whereas in cancer, desaturase metabolism is coupled with an elongation, to provide fatty acid components for cell membrane phospholipids. This difference of cell lipidome phenotype will be explored in further studies on larger groups, also in view of characterization of the existing molecular links between obesity and cancer [49,50], as well as with genetic variations in lipid desaturases [51].

The second aspect is connected to the inflammatory signaling, which is important in activating cancer proliferation pathways and resistance. This is sustained by the *n-*6 PUFA pathway and balanced by the omega-3 signaling pathway. Our study first ascertained the PUFAs supplementation according to the FFQ, determining that the levels of all omega-3 fatty acids and arachidonic acid intake (see Table 2), were higher in the cancer group. However, the levels of these fatty acids in cell membranes after adjustment of age, nutrient intake and BMI, were not found to differ between cancer patients and healthy controls (Table 3). In our group of cancer patients, the *n-*6 LA and DGLA showed higher levels compared to the control group, which remained significant also after adjustment of confounding factors. It is important to remark that DGLA is not relevantly present in foods, therefore it can be considered a “pure” metabolic indicator to be followed up in cancer patients [52].

In view of some studies addressing the changes in nutritional habits after diagnosis, towards healthier habits [53], we could estimate that also in our study, the cancer group changed the intake patterns into better diets richer in olive oil, oily and lean fish and with lower intakes of simple sugars and alcohol than controls. How the intake of *n-*3- and *n-*6 PUFAs on the lipid profile of mature RBC membrane can be evidenced with LA, which is significant after adjustment, whereas EPA and AA level changes are not significant in adjusted profiles by ANCOVA analysis. On the contrary, the dietary intake of FAs, such as oleic acid, had no effect in membrane FA composition as corroborated in other studies [54]. The elimination of confounding factors, such as diet was expedient to discriminate the metabolic differences in the lipid profiles between cancer patients and healthy subjects. Our study can be useful also in the debate on the use of diet and supplements in cancer prevention where there is a lack of sufficient safety and efficacy data [53].

We used a personalized molecular approach based on the RBC membrane lipid profile, to ascertain the presence of two main metabolic signatures of increased desaturation activity and omega-6 in cancer patients, that are also relevant targets of novel chemotherapeutical interventions [55,56]. Some final considerations must be evidenced: The first is the inclusion of several cancer types, that makes difficult to draw conclusion regarding the mechanisms; the second is that the membrane fatty acid analysis is referred to a cluster, but it should be obtained a consensus on the number of fatty acids describing the membrane status; the third is that this is an exploratory study to test the idea that membrane lipids in cancer patients can be independent from diets, and must be confirmed by population studies.

## 5. Conclusions

Despite the potentially important roles of diet and nutrition in cancer prevention and during the disease, there has been little evidence supporting a direct mechanistic link between food intake and disease. The results of the present study revealed that cancer patients present an altered lipid profile of mature membrane RBC independent from their dietary habits, as shown by a robust statistical treatment of the data. Using the approach of fatty acid-based membrane lipidomics, practical information can be obtained about the molecular characteristics of cancer patients, to be further confirmed in larger cohorts and development for improved patient management guidelines.

## Figures and Tables

**Table 1:** Characteristics of the population.

	Controls	Patients	*p*-value
	*n* (%)	*n* (%)	
Gender			0.578
Men	9 (24)	16 (30)	
Women	28 (76)	38 (70)	
Age (years) (median, Q1–Q3)	42 (32–57)	59 (51–64)	<0.001
BMI (kg/m^2^) (mean ± SD)	22.7 ± 4.0	24.6 ± 4.7	0.124
Cancer diagnosis			
Breast	-	20 (37)	-
Colon	-	13 (24)	-
Lung	-	9 (17)	-
Prostate	-	4 (7)	-
Ovarian	-	2 (4)	-
Lymphoma	-	1 (2)	-
Others	-	5 (9)	-
Chemotherapy regimen			
Paclitaxel	-	10 (19)	-
Oxaliplatin-based regimens	-	12 (22)	-
Docetaxel	-	4 (8)	-
Carboplatin	-	6 (11)	-
Anthracyclines (doxorubicin, TAC, AC, CHOP)	-	5 (9)	-
Cisplatin (with pemetrexed or gemcitabine)	-	6 (11)	-
5-Fluorouracil	-	2 (4)	-
Vinorelbine	-	4 (7)	
Others	-	5 (9)	-

Genders *p*-value was calculated with Chi-square test. Age is expressed for each group with median and first and third quartiles as it was not normally distributed. Its *p*-value is the result of U Mann-Whitney test. BMI (Body mass index), TAC (Taxotere, Adriamycin and Cyclophosphamide), AC (Adriamycine and Cyclophosphamide), CHOP (Cyclophosphamide, Adriamycin, Vincristine, Prednisolone).

**Table 2:** Nutrients and food intake in cancer and control groups.

	Total Nutrient/Food Intake		
	Control	Cancer	*p*
**Daily energy and nutrient intake**			
Calories (Kcal/day)	1716 (1475–2052)	1568 (1306–1989)	0.304
Carbohydrates (% of energy/day) *	37 (35–42)	35 (33–39)	0.025
Simple sugars (g/day)	90 (74–106)	42 (26–57)	<0.001
Protein (% of energy/day)	18 (16–19)	19 (17–21)	0.142
Fiber (g/day) *	25 (21–27)	17 (12–22)	<0.001
Alcohol (g/day) *	6 (2–10)	1 (0–5)	<0.001
Fat (% of energy/day)	39 (33–42)	42 (39–45)	0.006
Total SFA (% of energy/day)	11 (9–12)	11 (9–13)	0.773
C14:0 (g/day)	1.6 (1.1–2.0)	1.4 (0.7–2.2)	0.541
C16:0 (g/day) *	12 (9–14)	9 (7–12)	0.004
C18:0 (g/day) *	4.4 (3.7–6.2)	3.4 (2.2–4.6)	<0.001
Total MUFA (% of energy/day)	5 (5–8)	7 (6–8)	0.011
C16:1 (g/day) *	1 (0.8–1.3)	1.1 (0.8–1.5)	0.547
C18:1 (g/day) *	34 (29–36)	33 (26–39)	0.771
Total PUFA (% of energy/day) *	5.4 (4.7–7.6)	6.6 (5.8–7.8)	0.008
Total Omega 3 (% of energy/day) *	1.0 (0.9–1.2)	1.2 (1.0–1.4)	0.004
C18:3 (g/day) *	1.6 (1.3–2.5)	1.1 (0.8–1.5)	<0.001
C20:5 (g/day) *	0.09 (0.05–0.13)	0.30 (0.29–0.31)	<0.001
C22:5 (g/day) *	0.05 (0.03–0.07)	0.09 (0.08–0.10)	<0.001
C22:6 (g/day) *	0.24 (0.17–0.31)	0.58 (0.55–0.61)	<0.001
Total Omega 6 (% of energy/day) *	4.3 (3.6–6.2)	5.2 (4.3–5.9)	0.062
C18:2 (g/day) *	9 (7–14)	9 (7–12)	0.633
C20:4 (g/day) *	0.11 (0.08–0.13)	0.13 (0.11–0.15)	0.024
**Food groups (g/day)**			
Oily fish *	250 (117–250)	500 (500–500)	<0.001
Lean fish *	250 (250–500)	500 (500–500)	0.001
Shellfish *	93 (0–93)	50 (0–138)	0.934
Olive Oil *	175 (70–175)	245 (105–245)	<0.001
Nuts *	73 (35–275)	75 (0–131)	0.081
Fruit *	2800 (1493–3200)	2800 (800–4200)	0.478
Vegetables *	1600 (1200–2000)	1400 (925–1950)	0.161
Dairy products *	50 (0–425)	700 (313–1384)	<0.001
Eggs *	138 (138–207)	173 (138–276)	0.265
Red meat *	150 (70–220)	113 (0–300)	0.241

Data is expressed with medians and quartile 1 and quartile 3. * Not normally distributed variables. SFA, saturated fatty acid. MUFA, monounsaturated fatty acid. PUFA, Polyunsaturated fatty acid.

**Table 3:** Red blood cell (RBC) membrane fatty acid (FA) levels in cancer and control groups.

RBC Membrane FA (% rel)	Unadjusted Control	Unadjusted Cancer	*p*	Adjusted Control	Adjusted Cancer	*p*	Difference
	(*n* = 37)	(*n* = 54)		(*n* = 37)	(*n* = 54)		(%)
Saturated fatty acids							
Palmitic acid (16:0)	26.0 ± 0.2	24.6 ± 0.2	<0.001	26.1 ± 0.3	24.6 ± 0.2	0.003	−5.7
Stearic acid (18:0)	21.0 ± 0.2	17.4 ± 0.1	<0.001	20.8 ± 0.3	17.5 ± 0.2	<0.001	−15.9
Monounsaturated fatty acids							
Palmitoleic acid (16:1 *n-*7) *	0.4 ± 0.0	0.4 ± 0.0	0.506	0.4 ± 0.1	0.4 ± 0.1	0.305	-
Oleic acid (18:1 *n-*9) *	15.5 ± 0.2	17.6 ± 0.2	<0.001	15.5 ± 0.3	17.5 ± 0.2	<0.001	12.9
18:1 *n-*11 *	1.1 ± 0.0	1.7 ± 0.0	<0.001	1.1 ± 0.1	1.7 ± 0.1	<0.001	54.5
Polyunsaturated acid							
Linoleic acid (18:2*n-*6)	11.5 ± 0.2	11.9 ± 0.2	0.140	10.8 ± 0.3	12.5 ± 0.2	<0.001	15.7
Dihomo-γ-linoleic acid (20:3) *	1.8 ± 0.1	2.2 ± 0.1	<0.001	1.8 ± 0.1	2.2 ± 0.1	0.006	22.2
Arachidonic acid (20:4) *	16.1 ± 0.2	16.9 ± 0.2	0.033	16.8 ± 0.4	16.4 ± 0.3	0.472	-
EPA (20:5) *	0.8 ± 0.0	1.0 ± 0.1	0.026	0.8 ± 0.1	0.9 ± 0.1	0.323	-
DHA (22:6)	5.8 ± 0.1	6.0 ± 0.2	0.517	5.9 ± 0.2	5.9 ± 0.2	0.894	-
Trans fatty acids							
Trans 18:1 *	0.2 ± 0.0	0.1 ± 0.0	<0.001	0.2 ± 0.0	0.1 ± 0.0	<0.001	−50
Trans 20:4 *	0.2 ± 0.0	0.1 ± 0.0	<0.001	0.2 ± 0.0	0.1 ± 0.0	<0.001	−50
Total fatty acids							
Total SFA	47.0 ± 0.4	42.0 ± 0.1	<0.001	46.9 ± 0.4	42.0 ± 0.3	ND	−10.4
Total MUFA	17.0 ± 0.2	19.7 ± 0.2	<0.001	17.1 ± 0.3	19.7 ± 0.2	<0.001	15.2
Total PUFA	36.0 ± 0.3	38.0 ± 0.2	<0.001	36.0 ± 0.4	38.0 ± 0.3	<0.001	5.3
Total Omega 6	29.4 ± 0.3	31.0 ± 0.2	<0.001	29.3 ± 0.4	31.0 ± 0.3	0.002	5.8
Total Trans *	0.4 ± 0.2	0.2 ± 0.0	<0.001	0.4 ± 0.0	0.2 ± 0.0	ND	
Fatty acid indexes							
Saturation Index	2.8 ± 0.0	2.1 ± 0.0	<0.001	2.8 ± 0.1	2.2 ± 0.0	ND	−27.3
Unsaturation Index	150.9 ± 1.4	156.9 ± 1.3	0.001	150.9 ± 1.9	156.8 ± 1.5	0.049	3.83
Peroxidation Index	135.8 ± 1.6	141.8 ±1.4	0.006	136.3 ± 3.3	141.5 ± 1.8	0.143	-
Inflammatory risk Index *	4.3 ± 0.3	4.7 ± 0.2	0.591	4.6 ± 0.3	4.7 ± 0.2	0.801	-
PUFA Balance	18.3 ± 0.5	18.3 ± 0.6	0.428	18.6 ± 0.7	18.1 ± 0.5	0.927	-
*n-*3 cardiovascular risk index	6.6 ± 0.2	7.0 ± 0.2	0.085	6.7 ± 0.3	6.9 ± 0.2	0.699	-
Enzymatic indexes							
Δ9D 18:0/18:1 *	1.4 ± 0.0	1.0 ± 0.0	<0.001	1.4 ± 0.1	1.0 ± 0.0	<0.001	−28.6
Δ6D+ELO 18:2/20:3	6.7 ± 0.2	5.7 ± 0.2	0.001	6.4 ± 0.3	5.9 ± 0.2	0.219	-
Δ5D 20:4/20:3	9.3 ± 0.3	8.2 ± 0.3	0.009	10.0 ± 0.5	7.7 ± 0.4	0.002	−23.0
Δ9D 16:0/16:1 *	64.9 ± 2.7	59.0 ± 2.6	0.081	67.6 ± 4.0	57.1 ± 3.0	0.082	

Data are expressed with means ± standard error (se). Adjusted results are FA levels controlled by age, nutrient intake and BMI. * Not normally distributed variables. EPA (eicosapentaenoic acid), DHA (Docosahexaenoic acid), ELO (Elongase).
